# Systematic Review and Proportional Meta-Analysis of Endarterectomy and Endovascular Therapy with Routine or Selective Stenting for Common Femoral Artery Atherosclerotic Disease

**DOI:** 10.1155/2019/1593401

**Published:** 2019-04-14

**Authors:** Khalid Hamid Changal, Mubbasher Ameer Syed, Tawseef Dar, Muhammad Asif Mangi, Mujeeb Abdul Sheikh

**Affiliations:** ^1^Internal Medicine, Mercy Health St. Vincent Medical Center, Toledo, OH, USA; ^2^Cardiovascular Medicine, University of Toledo College of Medicine and Life Sciences, Toledo, OH, USA; ^3^Cardiology Division, Massachusetts General Hospital, Harvard Medical School, Boston, Massachusetts, USA; ^4^Cardiovascular Medicine and Interventional Cardiology, University of Toledo College of Medicine and Life Sciences, 3065 Arlington Ave. Toledo, OH 43614, USA

## Abstract

**Introduction:**

Common femoral endarterectomy (CFE) has been the therapy of choice for common femoral artery atherosclerotic disease (CFA-ASD). In the past, there was inhibition to treat CFA-ASD endovascularly with stents due to fear of stent fracture and compromise of future vascular access site. However, recent advances and new evidence suggest that CFA may no longer be a ‘stent-forbidden zone'. In the light of new evidence, we conducted a meta-analysis to determine the use of endovascular treatment for CFA-ASD and compare it with common femoral endarterectomy in the present era.

**Methods:**

Using certain MeSH terms we searched multiple databases for studies done on endovascular and surgical treatment of CFA-ASD in the last two decades. Inclusion criteria were randomized control trials, observational, prospective, or retrospective studies evaluating an endovascular treatment or CFE for CFA-ASD. For comparison, studies were grouped based on the treatment strategy used for CFA-ASD: endovascular treatment with selective stenting (EVT-SS), endovascular treatment with routine stenting (EVT-RS), or common femoral endarterectomy (CFE). Primary patency (PP), target lesion revascularization (TLR), and complications were the outcomes studied. We did proportional meta-analysis using a random-effect model due to heterogeneity among the included studies. If confidence intervals of two results do not overlap, then statistical significance is determined.

**Results:**

Twenty-eight studies met inclusion criteria (7 for EVT-RS, 8 for EVT-SS, and 13 for CFE). Total limbs involved were 2914 (306 in EVT-RS, 678 in EVT-SS, and 1930 in CFE). The pooled PP at 1 year was 84% (95% CI 75-92%) for EVT-RS, 78% (95% CI 69-85%) for EVT-SS, and 93% (95% CI 90-96%) for CFE. PP at maximum follow-up in EVT-RS was 83.7% (95% CI 74-91%) and in CFE group was 88.3% (95% CI 81-94%). The pooled target lesion revascularization (TLR) rate at one year was 8% (95% CI 4-13%) for EVT-RS, 19% (95% CI 14-23%) for EVT-SS, and 4.5% (95% CI 1-9%) for CFE. The pooled rate of local complications for EVT-RS was 5% (95% CI 2-10%), for EVT-SS was 7% (95% CI 3 to 12%), and CFE was 22% (95% CI 14-32%). Mortality at maximum follow-up in CFE group was 23.1% (95% CI 14-33%) and EVT-RS was 5.3% (95% CI 1-11%).

**Conclusion:**

EVT-RS has comparable one-year PP and TLR as CFE. CFE showed an advantage over EVT-SS for one-year PP. The complication rate is lower in EVT RS and EVT SS compared to CFE. At maximum follow-up, CFE and EVT-RS have similar PP but CFE has a higher mortality. These findings support EVT-RS as a management alternative for CFA-ASD.

## 1. Introduction

Atherosclerotic steno-occlusive disease of common femoral artery (CFA) in isolation is rare but often leads to symptomatic peripheral arterial disease [1]. In most instances, the atherosclerotic disease either extends beyond the CFA into contiguous arterial segments or is associated with multilevel disease proximally or distally. Historically, atherosclerotic steno-occlusive disease involving CFA is treated surgically with common femoral endarterectomy (CFE) which has so far remained the procedure of choice [2].

In the past, there was inhibition to treat common femoral artery atherosclerotic steno-occlusive disease (CFA-ASD) endovascularly with stents due to fear of stent fracture and compromise of future vascular access site. With significant advancements in the endovascular techniques, stent design, and adjunctive technologies, successful endovascular treatment of CFA-ASD is reported to be no longer considered a “stent forbidden zone” [2–7]. EVT of CFA disease has been shown to be associated with lower morbidity and mortality, shorter inpatient stay, improved patency, and faster recovery and in most cases requires local anesthesia only [2–7]. Paradigm shift to the treatment of CFA-ASD is limited by inconsistent results from earlier studies, mostly observational, examining the technical success and safety of EVT for treatment of CFA disease [2, 8, 9]. An older trial using bioabsorbable stents (BAS) showed worse one-year primary patency and increased redo procedures in the stent group compared to CFE [10]. Newer studies that are using more advanced endovascular techniques and stents are showing comparable clinical outcomes with EVT and CFE [11]. Thus, the role of EVT in CFA-ASD is still a subject of debate [3–8]. We henceforth conducted a meta-analysis to compare endovascular treatment for CFA-ASD with common femoral endarterectomy.

## 2. Materials and Methods

We conducted a thorough search of Pubmed, Medline, Cochrane, Embase, and clinicaltrials.gov to identify studies evaluating different treatment approaches of CFA ASD. The review was done per the Preferred Reporting Items for Systematic Reviews and Meta-Analyses (PRISMA) guidelines. A comprehensive search strategy, using an exhaustive list of MeSH terms, words, and synonyms, was carried out to identify the relevant studies on therapeutic approaches (endovascular and surgical) for CFA-ASD (Appendix 1, supplementary file). The bibliographies of relevant review articles and eligible studies were examined to complete a comprehensive search of all the published studies.

Inclusion criteria were randomized control trials, observational studies, and prospective or retrospective studies evaluating an endovascular treatment or CFE for CFA-ASD. Studies that measured outcomes including primary patency (PP) and/or target lesion revascularization (TLR) were included. Animal studies, studies with incomplete data, studies published before the year 2000, case reports, case series with less than 10 patients, and studies published in languages other than English were excluded from the analysis. In view of lack of an adequate number of RCTs, we performed a pooled proportional meta-analysis of outcomes for different therapeutic approaches from all the included studies on CFA-ASD.

**Figure 1:**
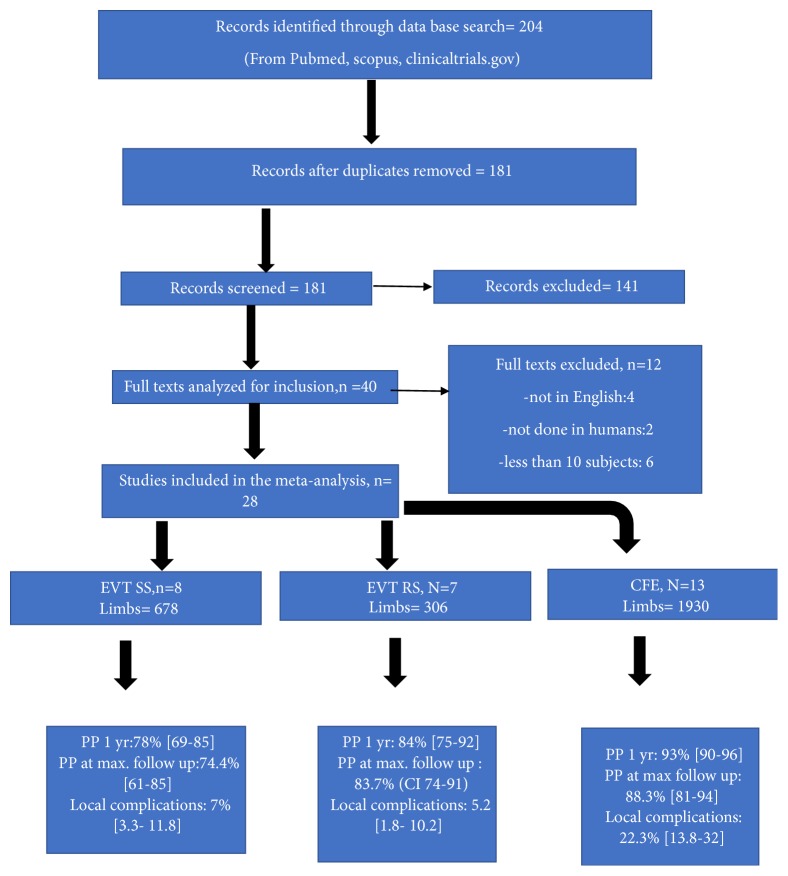
Flowsheet summarizing the selection of studies and main results.

We divided the patients in the studies into three groups based on the treatment strategy used for CFA-ASD: endovascular treatment with selective stenting (EVT-SS), endovascular treatment with routine stenting (EVT-RS), and common femoral endarterectomy [CFE] (Figure 1). In EVT-SS group primary treatment strategy was angioplasty only and stents were used only selectively. In EVT-RS group stenting with angioplasty was the primary treatment strategy. In CFE group endarterectomy of the common femoral artery was the primary treatment strategy and selected patients got additional endovascular treatment proximal or distal to CFA. CFE involves surgically opening the CFA longitudinally and peeling of the atherosclerotic plaque and in most cases involves patch angioplasty to close the vessel. PP, TLR, complications, and mortality were the main outcomes analyzed.

### 2.1. Data Collection

Two independent reviewers did the data collection [KC & SM]. Conflicts were sorted out by discussion and adjudication by a third reviewer [TD]. We did an analysis of search results for inclusion and exclusion criteria before data extraction. Multiple key variables were extracted including but not limited to author, year of publication, country where study was done, design of study, total duration of study, demographics and comorbidities, clinical presentation (claudication vs. critical limb ischemia), anatomy of lesions, types of stents used, length of follow-up, patency at follow-up, and complications. If we found duplicate studies, the most complete and latest article reporting relevant outcomes of interest was included. When articles did not report the number of limbs involved, we assumed them to be the same as the number of patients in the study. For the two randomized controlled trials included in the study, data about CFE and stenting was extracted separately into respective groups.

### 2.2. Statistical Analysis

Primary patency and complication rates were treated as dichotomous variables with, respectively, 95% confidence interval [CI]. Proportional meta-analysis was done using a random-effects model due to variations among the included studies and numerous uncontrolled variables. Forest plots were used to summarize the results. Each horizontal line in the forest plot represents a study. The length of this horizontal line represents 95% CI of the corresponding study's effect estimate. The effect estimate is marked by a solid black square on the horizontal line, the size of which corresponds to the weight a study exerts in the meta-analysis. The overall pooled estimate is represented by the diamond shape at the bottom of each forest plot. A horizontal line through the diamond stands for the CI of pooled results. The statistical significance between different interventions was defined if their corresponding 95% CI did not overlap. I^2^ statistic was used to assess statistical heterogeneity with an I^2^ > 50% regarded as statistically significant. A p < 0.05 was considered statistically significant for calculation of heterogeneity. This afforded a better measure of the consistency between the studies [35]. To assess publication bias we used Funnel plots performed by Egger tests. Asymmetric funnel plots are suggestive of publication bias.

## 3. Results

### 3.1. Study and Sample Characteristics

The search was performed from January 2000 to October 2017. After screening titles, abstracts, and excluding duplicate studies we obtained full-text copies of 167 studies relevant to EVT-SS, EVT-RS, or CFE for CFA-ASD. A total of 28 studies met all inclusion criteria (7 for EVT-RS, 8 for EVT-SS and 13 for CFE). Two RCTs had data on EVT-RS vs CFE and these were included to extract data into EVT-RS and CFE groups. Total of 2684 patients were included in the study (289 in EVT-RS, 646 in EVT-SS, and 1749 in CFE). The total number of limbs involved was 2914 (306 in EVT-RS, 678 in EVT-SS, and 1930 in CFE) [Figure 1].

### 3.2. Demographics and Comorbidities

Baseline demographics and comorbidities for the three study groups are given in Table 1. Men formed the majority in all study groups (61.7% in EVT-SS, 68.8% EVT-RS, and 69.9% in CFE). Hypertension was the most common comorbidity in all the study groups (84.9% in EVT-SS, 83.3% EVT-RS, and 85.6% in CFE). Most of the comorbidities and risk factors were distributed evenly amongst the three groups with a few exceptions. Coronary artery disease was less prevalent in the EVT-RS group (55.2% in EVT-SS, 36.3% EVT-RS, and 60.8% in CFE) whereas the EVT-SS group had fewer smokers (45.3% in EVT-SS, 59.1% EVT-RS, and 61.1% in CFE) but more patients with ESRD (27.5% in EVT-SS, 17.4% EVT-RS, and 11.8% in CFE).

### 3.3. Clinical Presentation

Clinical presentation was classified by Fontaine Classification [36] in some publications and by Rutherford Classification [37] in others. After reviewing data of all the included patients in the three treatment groups, we reclassified them either as Claudication [Fontaine Stagse I & II | Rutherford Categories 0, 1, 2, and 3] or CLI [Fontaine Stages III and IV | Rutherford Categories 4, 5, and 6] patients. The distribution of patients presenting with claudication versus CLI was 64.8% vs 35.1% in EVT-SS, 66.1% vs 33.5% in EVT-RS, and 53.7% vs 58.1% in CFE, which revealed that the patients in CFE group could have had more advanced disease in comparison to EVT groups (Table 1).

### 3.4. Lesion Characteristics

In the published studies various classification systems were used for describing lesion characteristics. We reviewed two classification systems described previously for CFA-ASD. Bonvini et al. [38] described CFA lesions based on Medina Classification that is used for bifurcation coronary artery disease. The second classification system described by Azema et al. [12] is based on inflow and outflow lesions and classifies CFA-ASD into 4 categories. We chose the latter for reclassifying the lesions in various treatment groups as it was more convenient to apply and easier to correlate with outcomes.  Table 2 details the lesion types included in each treatment group. Isolated CFA lesions categorized as type II lesions were seen in 47.5%, 30.3%, and 42.9% in EVT-SS, EVT-RS, and CFE groups, respectively.

### 3.5. Procedural Data

Total of 330 CFA stents were placed in EVT-RS group and majority were self-expanding stents (71.8%). In EVT-SS group 123 CFA stents were placed, with 86.4% stents being self- expanding. A total of 552 stents were placed in CFE group during surgery, all of which were either proximal or distal to CFA. In EVT-RS group 24 stents were repunctured without any complication. Two stents were repunctured in EVT-SS group. In the endovascular group most procedures were performed by gaining the access in a contralateral cross-over fashion (70.6% in EVT-SS and 78.1% in EVT-RS). Ipsilateral retrograde approach was used in 15.3% in EVT-SS and 12.4% in EVT-RS. In the CFE group, 48.9% (944) procedures were CFE only, while 41.1% (794) were hybrid procedures involving an additional endovascular procedure. Patch angioplasty was used in 72.1% (13 out of 14) whereas 5.6% (n=110) patients underwent concomitant IFBP (ilio-femoral-bypass-grafting). The procedural details for the three groups are illustrated in Table 3.

### 3.6. Clinical Efficacy

**Figure 2:**
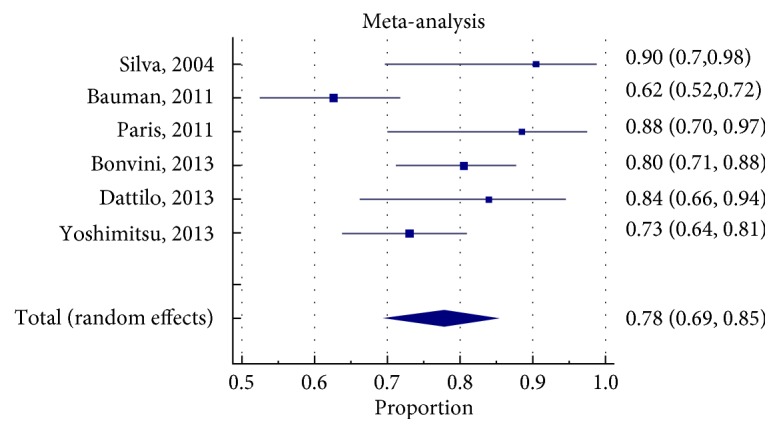
Forrest plot for PP (Primary patency) at 1 year in EVTSS (Endovascular therapy with selective stenting) group.

**Figure 3 fig3:**
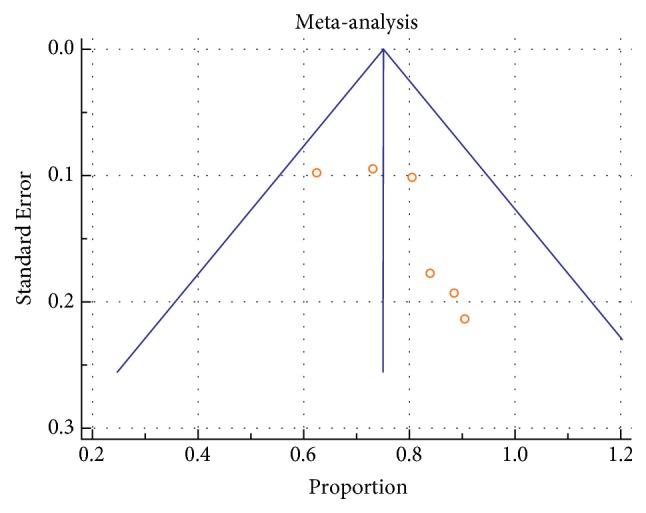
Funnel plot of studies regarding PP at 1 year in EVT SS by Egger test: asymmetrical.

**Figure 4 fig4:**
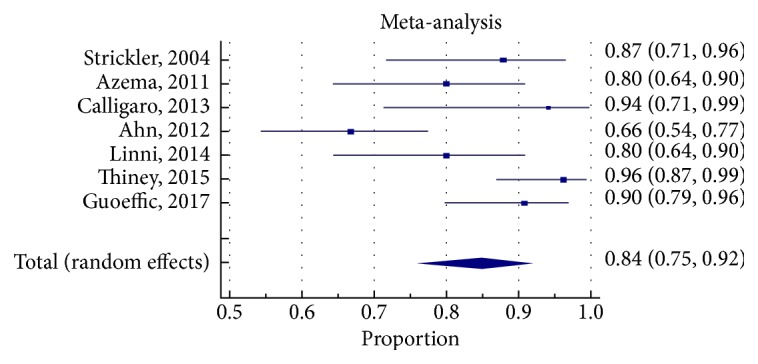
Forest plot for primary patency at 1 year in EVT RS group.

**Figure 5 fig5:**
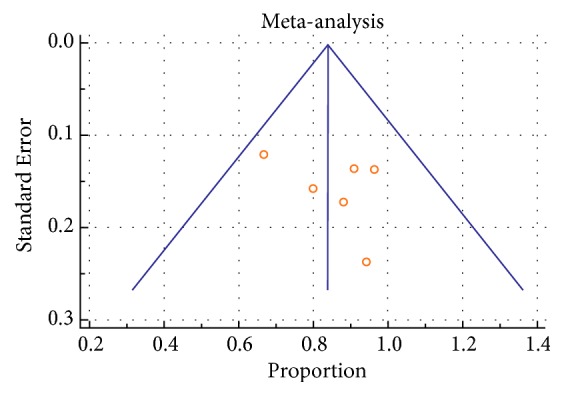
Funnel plot of studies regarding PP at I year in EVTRS group by Egger test. Symmetrical.

**Figure 6 fig6:**
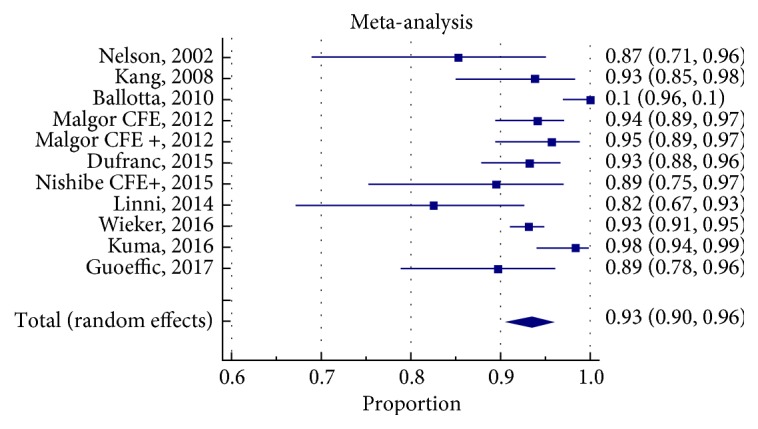
Forrest plot for PP at 1 year in CFE group.

**Figure 7:**
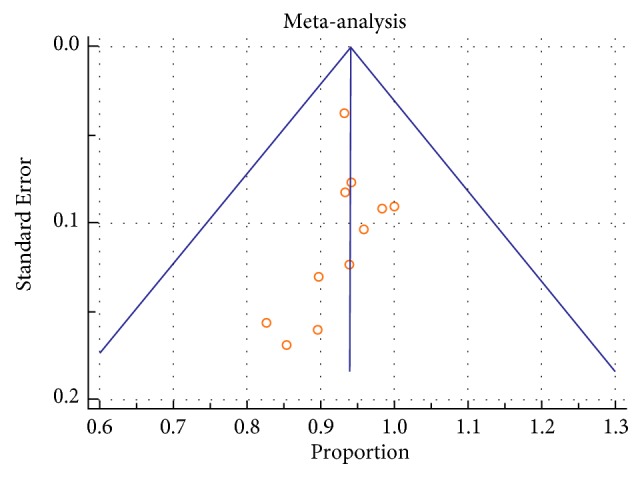
Funnel plot of studies about PP at I year in CFE group by Egger test: asymmetrical.

We used primary patency and target vessel revascularization rates (as defined in Appendix 2) to be the measures of clinical efficacy for the different treatment strategies. The pooled proportion for primary patency (PP) at 1 year was 78% (95% CI 69-85%) for EVT-SS group, 84% (95% CI 75-92%) for EVT-RS group, and 93% (95% CI 90-96%) for CFE group (Figures 2 [Fig fig3] [Fig fig4] [Fig fig5] [Fig fig6]–7). The CIs of EVT-RS and CFE groups overlap and thus statistically significant difference could not be proven between these two treatment strategies. On the other hand, CFE showed clear advantage over selective stenting strategy [EVT-SS] in terms of PP at 1 year. The pooled target lesion revascularization (TLR) rates at one year were 8% (95% CI 4-13%) for EVT-RS, 19% (95% CI 14-23%) for EVT-SS, and 4.5% (95% CI 1-9%) for CFE (supplementary file Figures 8-13). The CIs of CFE and EVT RS overlap while CI of EVT SS does not overlap with either EVT RS or CFE. Hence while superior to selective stenting, both routine stenting and endarterectomy had comparable rates of target lesion revascularization at 1 year.  Table 4 summarizes the results elaborated above.  Table 5 describes the studies that were included in the meta-analysis.

PP at maximum follow-up in EVT-RS was 83.7% (95% CI 74-91%) (Figure 14 supplementary file), in CFE group was 88.3% (95% CI 81-94%) (Figure 15). Thus, the PP was comparable in the two groups. The maximum follow-up was similar in the EVT-RS and CFE groups but much lower in the EVT-SS group and thus not amenable for comparison. The average maximum follow in EVT-RS was 66.9 months (range 28-158), EVT-SS was 32.1 (range 16-36), and CFE was 80.01 (range 19-168).

### 3.7. Mortality and Complication Rates

The pooled mortality at 30 days was 0.8% (95% CI 0.1-2%) for EVT-RS, 1% (95% CI 0.4- 2%) for EVT-SS, and 1.3% (95% CI 0.6-2%) for CFE (supplementary file Figures 16-21). There was no statistically significant difference between the treatment strategies in this regard as CIs of the three groups overlap. The pooled rate of local complications for EVT-RS group was 5% (95% CI 2-10%) while the EVT-SS had 7% local complication rates (95% CI 3.3 to 11.8%) and CFE had a pooled local complication rate of 22% (95% CI 14-32%) (supplementary file Figures 22-27). The pooled rate of amputations was 3% (95% CI 1-6%) for EVT-RS, 4% (95% CI 2.4 to 6.0%) for EVT-SS, and 4.5% (95% CI 2.5 to 6.8%) for CFE (supplementary file Figures 28-33) (Tables 4 and 6).

Mortality at maximum follow-up in CFE group was 23.1% (95% CI 14-33%) and EVT-RS was 5.3% (95% CI 1.6-11%) (Figures 34-36, supplementary file). Thus, mortality at maximum follow-up is much smaller (statistically significant) in EVT-RS than CFE. As mentioned above, the maximum follow-up was similar in the EVT-RS and CFE groups but much lower in the EVT-SS group.

### 3.8. Heterogeneity

Heterogeneity between the included studies using I^2^ was documented for PP statistics at 1 year in all groups (EVTSS 69.4%, p= 0.006, EVT RS 75.5%, p=0.0004, CFE 74.9%, and p < 0.0001). Heterogeneity in studies was not significant for TLR at one year in EVT SS (22.3%, p = 0.27) and EVTRS (38.7%, p = 0.16), while it was significant in CFE (85.1%, p < 0.0001). Heterogeneity in studies was not significant for data on amputations in EVT SS (14.5%, p=0.31) and EVTRS (0.36%, p=0.4), while it was significant in CFE (75.3%, p< 0.0001). Heterogeneity in studies was not seen for 30 days of mortality in any group (EVTSS 0%, p=0.43, EVTRS 0%, and p=0.9; CFE 0%, p=0.56). Heterogeneity between the included studies using I^2^ was documented in local complications in all groups (EVTSS 77.1%, p=0.0001, EVTRS 56.9%, p=0.04, CFE 95.2%, and p < 0.0001) (Table 1). Funnel plots were used to assess for publication bias. Asymmetric funnel plots were seen in EVTSS group for PP at 1 year (Figure 3) and TLR at one year (Figure 9, supplementary file) showing possible publication bias. Asymmetric funnel plots were not seen in EVTRS group. Asymmetric funnel plots were seen in CFE group for PP at one year (Figure 7), amputations (Figure 33, supplementary file), and local complications (Figure 27, supplementary file) possibly due to publication bias.

## 4. Discussion

In our analysis, one-year PP in EVT-RS and CFE was similar (EVT-RS 84%, 95% CI 75- 92%; CFE 93%, and 95% CI 90-96%). The rate of local complications was higher in CFE (22%, 95% CI 14- 32%) compared to either EVT-RS (5%, 95% CI 2-10%) or EVT-SS (7%, 95% CI 3- 11%). Hence, endovascular therapy with routine stenting had similar PP at one year as compared to CFE but had lesser complications. However, both CFE and EVT-RS had better one-year PP than EVT-SS 78% (95% CI 69-85%). Thus, selective stenting of CFA is not a good treatment strategy. At maximum follow-up CFE and EVT-RS continued to have similar PP. However, the mortality in the CFE group was significantly higher than in the EVT-RS group.

Our results are similar to the results of TECCO trial [(Traitement des Lésions Athéromateuses de l'Artère Fémorale Commune par Technique Endovasculaire Versus Chirurgie Ouverte (Endovascular Versus Open Repair of the Common Femoral Artery))] which compared EVT-RS with CFE. TECCO trial showed that there was no significant difference in primary patency (one-year PP in CFE-90%, EVT-90%), sustained clinical improvement, and target revascularization rates between CFE and EVT-RS groups. The rate of perioperative complications (26% in CFE vs. 12.5% in EVT) and hospital stay (6.3 ± 3 in CFE vs.3.2 ± 2.9 in EVT) was more in the CFE group compared to EVT-RS group. Self-expanding nitinol stents were used in this study [11]. The only other RCT comparing EVT-RS and CFE was published by Linni et al. in 2014 [10]. While the patency rates were significantly lower in the stented group compared to CFE (one-year PP was 80% in BAS vs. 100% in CFE; p=0.007), the stents used in this study were bioabsorbable Poly L Lactic Acid stents that have struggled to demonstrate better outcomes in the coronary vasculature as well [22, 39–41]. The worse performance of BAS may be related to the lower radial strength of bioresorbable scaffolds and hence poor patency rates. The bioresorbable stent technology has shown inferiority in the coronary arteries in the Absorb III and AIDA trial where they failed to deliver better long-term outcomes in comparison to drug-eluting nitinol stents and were associated with increased stent thrombosis, including late stent thrombosis [22, 39–41].

Stent fractures, which are cited as a major problem with endovascular therapy of CFA, were reported in only 1.9% of the patient included in the routine stenting group. Twenty-four stents were repunctured for vascular access in the routine stenting group; no complications were reported. This shows that stent fracture in CFA location is uncommon and concerns regarding later access may not be a reason to exclude endovascular therapy as a possible procedure of choice in suitable candidates. In general, stent fractures are a result of stent design, the anatomy of the stented segment, biomechanical forces, and plaque morphology [42]. The design of stents used for CFA atherosclerotic disease has evolved. Majority of the stents deployed in the endovascular therapy groups were self-expanding. Percentage of self-expanding vs. balloon-expanding stents was 71.8% vs 28.2% in EVT-RS group whereas it was 86.4% vs 13.6% in EVT-SS. Self-expanding stents are largely replacing balloon expanding stents in lower limb arterial disease. Most self-expanding stents are now made of nitinol which is an alloy of nickel and titanium. An important feature of nitinol that is beneficial in the self-expanding design of stents is its thermal shape memory and super-elasticity [10, 36, 42–44]. On the contrary, stiffer balloon-expandable stents have less radial strength and high chances of deformation and fracture [45].

As described previously CFE had more complications than EVT-RS or EVT-SS. The most common complications reported in EVT-RS group were in-stent restenosis (10.4%), stent fracture (1.9%), hematoma (0.65%), inflow/outflow vessel stenosis (0.65%), stent occlusion (0.65%), and thrombosis (0.32%) [Table 6]. The most common complications in EVT-SS group were hematomas (3.2%), thromboembolic complications (1.17%), and perforation/dissection (0.44%). While local wound infections are practically non-existent in the EVT groups, it was the most common complication encountered in CFE (7.3%) followed by cardiac complications (including myocardial infarction) (4.58%), inguinal lymph leaks (2.5%), hematoma/bleeding (1.55%), and acute renal failure (1.24%). Systemic complications were more common in the CFE group. The mean age of patients was 68.4 years in EVT-SS group, 68.8 years in EVT-RS group, and 69.9 years in CFE group. Majority of patients were men in our analysis (61.7% in EVT-SS, 68.8% EVT-RS, and 69.9% in CFE; Table 1) and based on prior epidemiology studies peripheral arterial disease is more common in men as compared to women [41, 46].

## 5. Limitations

Only two RCTs were available for comparison. One used bioabsorbable stents which are not effective stents. Ideally, a meta-analysis of RCTs would be best but due to nonavailability of RCTs on the current topic, a proportional meta-analysis provides the best evidence. There was significant heterogeneity in EVTSS group for PP, primary assisted patency, and local complications. The funnel plots for PP and TLR are asymmetric possibly due to publication bias. Heterogeneity was also seen for PP, primary assisted patency and local complications in EVTRS group. The funnel plots in EVT RS group were symmetric showing less likelihood of publication bias. Heterogeneity was seen in CFE studies for PP, primary assisted patency, TLR, amputations, and local complications. Funnel plots are asymmetrical for primary patency, primary assisted patency, amputations, and local complications from possible publication bias. Overall, the heterogeneity was possibly due to lack of consistency in the design of studies, patient selection and reporting of complications. The higher percentage of CLI patients in CFE group (58.1%) than in EVT-selective stenting (35.1%) or EVT-routine stenting (33.5%) could have affected the outcomes studied in this study. A study matched for CLI is lacking currently in literature and if done may give more accurate outcomes.

## 6. Conclusion

Endovascular therapy with routine stenting strategy has comparable primary patency and target lesion revascularization rates as compared to common femoral endarterectomy. Complications and mortality of CFE are higher than EVT. These findings support endovascular therapy as an alternative in suitable candidates. There is a need for large randomized controlled trials to compare outcomes of routine stenting strategy to common femoral endarterectomy.

## Figures and Tables

**Table 1:** Baseline demographics and comorbidities of patients in the different treatment groups.

Variable	EVT - Selective Stenting	EVT- Routine Stenting	CFE
	n (%)	n (%)	n (%)
Total studies	8	7	14

Number of patients	646	289	1756

Number of limbs	678	306	1930

Mean Age [years]	68.4	68.8	69.9

Males	399 (61.7)	202 (69.8)	1299 (73.9)

Females	247 (38.3)	87 (30.2)	457 (26.1)

Diabetes Mellitus	253 (39.1)	102 (35.2)	736 (41.9)

Coronary Artery Disease	357 (55.2)	105 (36.3)	1069 (60.8)

Hypertension	451/531 ^#^ (84.9)	241 (83.3)	1504 (85.6)

Hyperlipidemia	317/531 ^#^ (59.6)	183 (63.3)	1106/1666 ^*∗*^ (66.3)

Smokers	241/531 ^#^ (45.3)	171 (59.1)	1074 (61.1)

End Stage Renal Disease	84/305 ^@^ (27.5)	19/109 (17.4)^¶^	164/1379 ^∧^ (11.8)

Claudication	419 (64.8)	191 (66.1)	943 (53.7)

CLI	227 (35.1)	97 (33.5)	1021 (58.1)

[@ 3 out of 8 studies | # 7 out of 8 studies |  *∗* 12 out of 13 studies |  ∧ 9 out of 13 studies].

**Table 2:** Classification of lesions included in the different treatment groups.

Variable	EVT - Selective Stenting	EVT- Routine Stenting	CFE
	n (%)	n (%)	n (%)
Type I Lesion [Inflow + CFA]	84/574 ^#^ (14.6)	63 (20.5)	318/844 ^$^ (37.6)

Type II Lesion [Isolated CFA]	310/652 ^#^ (47.5)	93 (30.3)	716/ 1668 ^*ƍ*^ (42.9)

Type III Lesion [Outflow + CFA]	265/574 ^#^ (46.1)	125 (40.8)	410/955 ^Ω^ (42.9)

Type IV Lesion [Bypass grafts and other lesions]	26 (3.8)	25 (8.2)	0

[**#** 7 out of 8 studies |  $ 10 out of 13 studies |  *ƍ* 12 out of 13 studies |  ^Ω^ 11 out of 13 studies].

**Table 3:** Procedural details in different treatment groups.

Variable	EVT - Selective Stenting	EVT- Routine Stenting	CFE
	n (%)	n (%)	n (%)
Total stents	123	330	552^∞^

Balloon-Expandable stents	15/110 (13.6) ^#^	93 (28.2)	DNA

Self-Expandable stents	95 /110 (86.4) ^#^	237 (71.8)	DNA

Bioabsorbable stents	0 (0)	41 (12.4)	DNA

Covered stents	0 (0)	17 (5.1)	DNA

Atherectomy	72 (10.6)	15 (4.9)	DNA

Contralateral cross-over approach	479 (70.6)	239 (78.1)	DNA

Ipsilateral retrograde approach	104 (15.3)	38 (12.4)	DNA

Stent Fracture	0 (0)	6 (1.8)	DNA

Stent Re puncture	2 (1.6)	24 (7.2)	DNA

CFE only	DNA	DNA	944 (48.9)

Hybrid CFE (CFE + endovascular procedure)	DNA	DNA	794 (41.1)

CFE + Distal Revascularization	DNA	DNA	401/1721 (23.3) ^*ƍ*^

CFE + Proximal Revascularization	DNA	DNA	495/1721 (28.7) ^*ƍ*^

CFE + Proximal and Distal Revascularization	DNA	DNA	81/1683 (4.8) ^*π*^

Patch angioplasty	DNA	DNA	1325/ 1837 (72.1) ^*∗*^

Profundaplasty	DNA	DNA	414/743 (55.7) ^*φ*^

Concomitant Bypass	DNA	DNA	110 (5.6)

[**#** 7 out of 8 studies |  *π* 11 out of 13 studies |  *ƍ* 12 out of 13 studies |  *∗* 12 out of 13 studies |  ∞ concomitant proximal or distal stents (not in CFA)] *DNA-Does not apply*.

**Table 4:** Clinical efficacy as demonstrated by pooled analysis of identified outcomes between the different treatment strategies.

Variable	EVT - Selective Stenting	EVT- Routine Stenting	CFE
Primary Patency at 1 yr [95% CI] [I^2^, p]	78% [69-85] [69.37%, p= 0.006]	84% [75-92] [75.52%, p=0.0004]	93% [90-96] [74.91%, p < 0.0001]

Primary Assisted Patency at 1 yr [95% CI] [I^2^, p]	86% [67-97] [91.8%, p < 0.0001]	94% [83-99] [81.29%, p = 0.0011]	97% [94-99] [90.87%, p < 0.0001]

Target Lesion revascularization at 1 yr [95% CI] [I^2^, p]	19% [14-23] [22.3%, p = 0.27]	8% [4-13] [38.76%, p = 0.1628]	4.5% [1-9] [85.07%, p < 0.0001]

Amputations [95% CI] [I^2^, p]	3% [1.3 to 5.3] [ 14.5%, p=0.31]	3% [1-6] [0.36%, p=0.4]	4.5 [2.5- 6.8] [75.32%, p< 0.0001]

30 days mortality [95% CI] [I^2^, p]	1% [0.4-2] [0%, p=0.43]	0.8% [0.1-2] [0%, p=0.9]	1.3% [0.6-2] [0%, p=0.56]

Local complications [95% CI] [I^2^, p]	7% [3.3 to 11.8] [77.08%, p=0.0001]	5.2 [1.8- 10.2] [56.89%, p=0.04]	22.3% [13.8-32] [95.2%, p < 0.0001]

An I^2^ more than 50% and a p less than 0.05 showing heterogeneity. Yr: year and CI: confidence interval.

**Table 5:** Showing the details of the studies included in the meta-analysis.

Study	YOP	Country	Design	Study Period	Total Duration in Years	Mean age
Stickler [9]	2004	Switzerland	Retrospective Analysis	1995-2002	7	70

Azema/Nasr [12, 13]	2011	France	prospective cohort	2006-2008	3	67.3

Calligaro [14]	2011	USA	Retrospective Analysis	2005-2010	6	69.5

Ahn [15]	2012	USA	Retrospective Analysis	2009-2011	3	67.2

Linni: Stent [10]	2014	Austria	RCT	2011-2013	2	71.6

Thiney [16]	2015	France	Retrospective Analysis	2009-2013	4	68

Guoeffic: Stent [11]	2017	France	Prospective cohort	2011-2013	2.5	68

EVT Selective Stenting group						

Silva [17]	2004	USA	retrospective review of prospectively managed database	na	na	64.9

Bauman [18]	2011	switzerland	retrospective review of prospectively managed database	1995-2009	15	72

Paris [19]	2011	USA	Retrospective Analysis	1994-2009	15 y	68.9

Bonvini [20]	2013	Germany	Retrospective Analysis	1996-2007	11	68.7

Dattilo [21]	2013	USA	Retrospective Analysis	2006-2011	6	62.8

Davies [22]	2013	UK	Retrospective Analysis	2006-2012	6 y	71

Yoshimitsu [23]	2013	Japan	Retrospective Analysis	2001-2010	10	71

Mehta [41]	2016	USA	prospectively maintained multicenter database	2006-2013	7	68

CFE group						

Nelson [24]	2002	USA	Retrospective Analysis	1997-2000	3	70.8

Kang [25]	2008	USA	retrospective review of prospectively gathered data	2002-2005	4	74

Chang [26]	2008	USA	Retrospective review	1997-2006	10	73.5

Kechegias [27]	2008	Finland	Retrospective Review	1983-2006	13	72.4

Ballotta [28]	2010	Italy	prospective cohort	2000-2007	8	68

Desai [29]	2011	UK	retrospective review	1996-2008	13	71.2

Malgor [30]	2012	USA	Retrospective Analysis	1997-2008	11	68

Dufranc [31]	2015	France	prospective cohort	2010-2012	3	68.7

Nishibe [32]	2015	japan	retrospective review	2010-2014	4	67

Linni [10]	2014	Austria	RCT	2011-2013	2	67.4

Wieker [33]	2016	Germany	retrospective 2 center	2006-2012	6	69.4

Kuma [34]	2016	japan	retrospective multicenter	1998-2014	17	68.6

Guoeffic [11]	2017	France	RCT	2011-2013	2.5	69.4

**Table 6:** Details of complications.

Complications reported in EVT Routine Stenting	
[n=306 limbs]	n [%]
Hematoma	2 (0.65)

Inflow/outflow vessel stenosis	2 (0.65)

Stent occlusion	2 (0.65)

Thrombosis	1 (0.32)

Vascular perforation	1 (0.32)

Local infection	1 (0.32)

Stroke	1 (0.32)

In stent restenosis	32 (10.4)

Strut failure	1 (0.32)

Stent fracture	6 (1.9)

Complications reported in EVT Selective Stenting	
[n= 678 limbs]	

Thromboembolic complications	8 (1.2)

Pseudo aneurysm	2 (0.29)

AV Fistula	1 (0.14)

Perforation/CFA dissection	3 (0.44)

Pulmonary edema	1 (0.14)

Myocardial Infarction	3 (0.44)

Retroperitoneal bleed	1 (0.14)

Groin hematomas	22 (3.2)

Acute Kidney Injury	1 (0.14)

Sepsis	1 (0.14)

Complications reported in CFE	
[n=1930 limbs]	

Wound/local infection	142 (7.3)

Myocardial infarction	21 (1.1)

Proximal external iliac artery dissection	1 (0.05)

Cardiac complications	69 (3.5)

Pulmonary complications	21 (1.1)

Thrombotic and embolic complications	9 (0.46)

Inguinal lymph leaks	49 (2.5)

Hematoma/bleeding comp	30 (1.5)

Seroma	6 (0.31)

Acute Kidney Injury	24 (1.2)

Neurological complications	10 (0.5)

Delayed wound healing	19 (0.98)_

Sepsis	4 (0.21)

## Data Availability

The data used to support the findings of this study are available from the corresponding author upon request.

## Supplementary Materials

Supplementary Materials Figure 8: Forrest plot for TLR 1 year in EVT SS group. Figure 9: Funnel plot for TLR 1 year EVT SS group: asymmetrical. Figure 10: Forrest plot for TLR at 1 year in EVT RS group. Figure 11: Funnel plot for TLR at 1 year in EVT RS group: symmetrical. Figure 12: Forrest plot for TLR at 1 year in CFE group. Figure 13: Funnel plot for TLR at 1 year in CFE group: symmetrical. Figure 14: Forrest plot for PP at maximum follow-up in EVT-RS: PP- 83.7% (95% CI 0.74-0.91). Figure 15: Forrest plot for PP in CFE at maximum follow-up: PP- 88.34% (95% CI 0.81-0.94). Figure 16: Forrest plot for mortality at 30 days in EVT SS group. Figure 17: Funnel plot for mortality 30 days in EVT SS group: symmetrical. Figure 18: Forrest plot for mortality 30 days in EVT RS group. Figure 19: Funnel plot for mortality at 30 days in EVT RS group: symmetrical. Figure 20: Forrest plot for mortality at 30 days in CFE group. Figure 21: Funnel plot for mortality at 30 days in CFE group: symmetrical. Figure 22: Forrest plot for local complications in EVTSS group. Figure 23: Funnel plot for local complications in EVT SS group: symmetrical. Figure 24: Forrest plot for local complications in EVT RS group. Figure 25: Funnel plot for local complications, EVTRS group: symmetrical. Figure 26: Forrest plot for local complications in CFE group. Figure 27: Funnel plot for Local complications in CFE group: asymmetrical. Figure 28: Forrest plot for amputations in EVT SS group. Figure 29: Funnel plot for amputations in EVT SS group: symmetrical. Figure 30: Forrest plot for amputations in EVT RS group. Figure 31: Funnel plot for amputations in EVT SS group: symmetrical. Figure 32: Forrest plot for amputations in CFE group. Figure 33: Funnel plot for amputations in CFE group: asymmetrical. Figure 34: Forrest plot for mortality at max follow-up in CFE group. Mortality was 23.1% (95% CI 0.14-0.33). Figure 35: Forrest plot for mortality at max follow-up in EVT-RS group. Mortality was 5.33% (95% CI 0.016-0.11). Figure 36: Forrest plot for mortality at max follow-up in EVT-SS group. Mortality was 10.9% (95% CI 0.03-0.22).
