# Supplementary material for: Systematic Review and Proportional Meta-Analysis of Endarterectomy and Endovascular Therapy with Routine or Selective Stenting for Common Femoral Artery Atherosclerotic Disease
Source: J Interv Cardiol. 2019 Apr 14;2019:1593401. doi: 10.1155/2019/1593401 (PMC6739799; doi:10.1155/2019/1593401)
Supplement: Supplementary Materials — Figure 8: Forrest plot for TLR 1 year in EVT SS group. Figure 9: Funnel plot for TLR 1 year EVT SS group: asymmetrical. Figure 10: Forrest plot for TLR at 1 year in EVT RS group. Figure 11: Funnel plot for TLR at 1 year in EVT RS group: symmetrical. Figure 12: Forrest plot for TLR at 1 year in CFE group. Figure 13: Funnel plot for TLR at 1 year in CFE group: symmetrical. Figure 14: Forrest plot for PP at maximum follow-up in EVT-RS: PP- 83.7% (95% CI 0.74-0.91). Figure 15: Forrest plot for PP in CFE at maximum follow-up: PP- 88.34% (95% CI 0.81-0.94). Figure 16: Forrest plot for mortality at 30 days in EVT SS group. Figure 17: Funnel plot for mortality 30 days in EVT SS group: symmetrical. Figure 18: Forrest plot for mortality 30 days in EVT RS group. Figure 19: Funnel plot for mortality at 30 days in EVT RS group: symmetrical. Figure 20: Forrest plot for mortality at 30 days in CFE group. Figure 21: Funnel plot for mortality at 30 days in CFE group: symmetrical. Figure 22: Forrest plot for local complications in EVTSS group. Figure 23: Funnel plot for local complications in EVT SS group: symmetrical. Figure 24: Forrest plot for local complications in EVT RS group. Figure 25: Funnel plot for local complications, EVTRS group: symmetrical. Figure 26: Forrest plot for local complications in CFE group. Figure 27: Funnel plot for Local complications in CFE group: asymmetrical. Figure 28: Forrest plot for amputations in EVT SS group. Figure 29: Funnel plot for amputations in EVT SS group: symmetrical. Figure 30: Forrest plot for amputations in EVT RS group. Figure 31: Funnel plot for amputations in EVT SS group: symmetrical. Figure 32: Forrest plot for amputations in CFE group. Figure 33: Funnel plot for amputations in CFE group: asymmetrical. Figure 34: Forrest plot for mortality at max follow-up in CFE group. Mortality was 23.1% (95% CI 0.14-0.33). Figure 35: Forrest plot for mortality at max follow-up in EVT-RS group. Mortality was 5.33% (95% CI 0.016-0.11). Figure 36: [file 1593401.f1.pdf]

SUPPLEMENTARY DATA

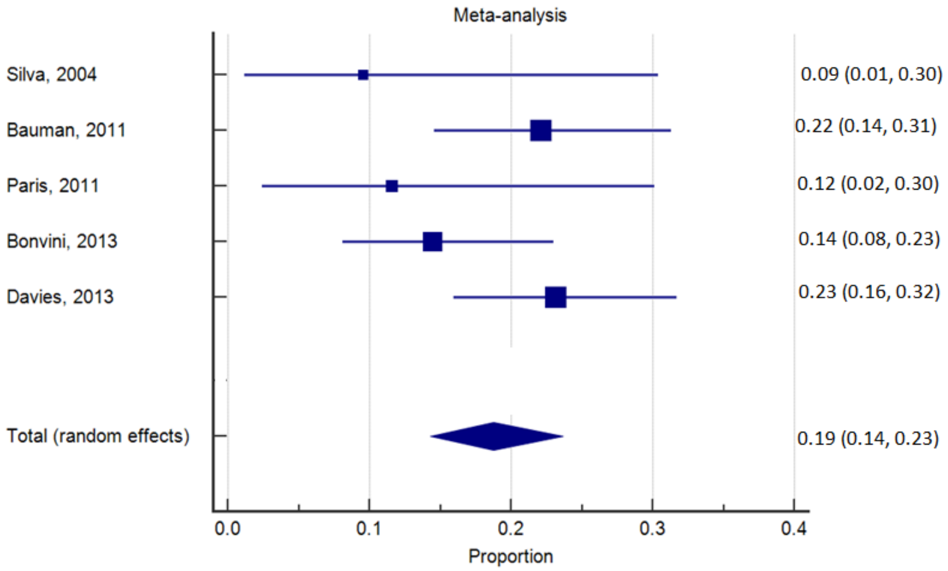

Figure 8: Forrest plot for TLR 1 year in EVT SS group.

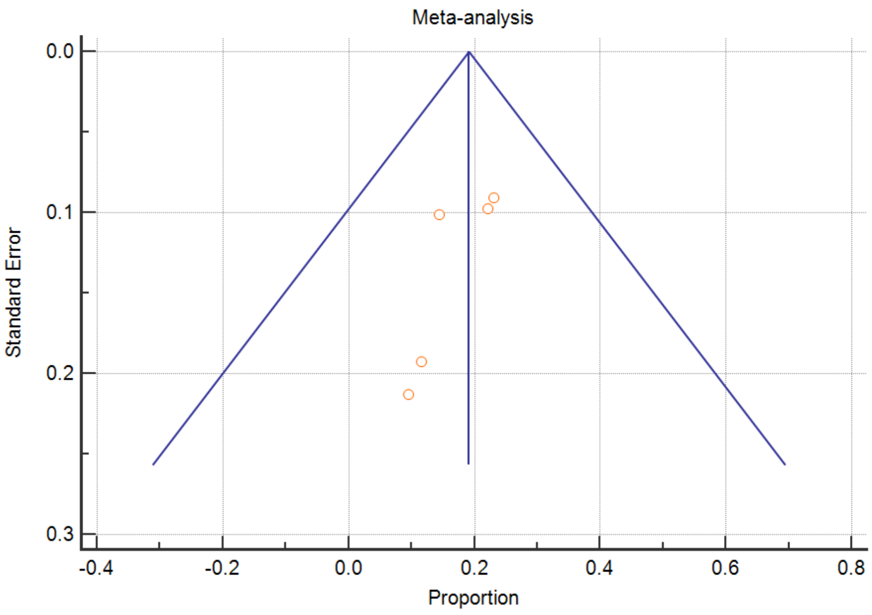

Figure 9: Funnel plot for TLR 1 year EVT SS group. Asymmetrical.

Meta-analysis: proportion

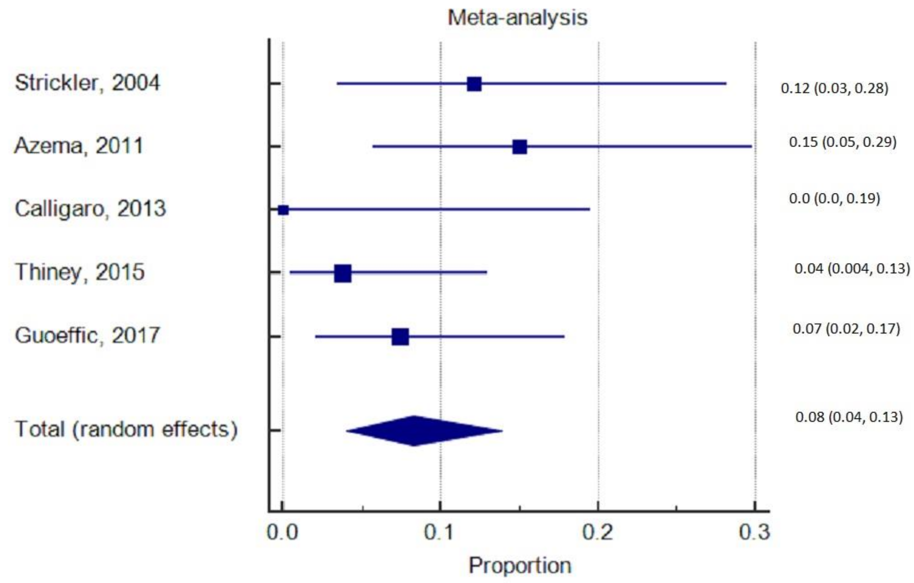

Figure 10: Forrest plot for TLR at 1 year in EVT RS group.

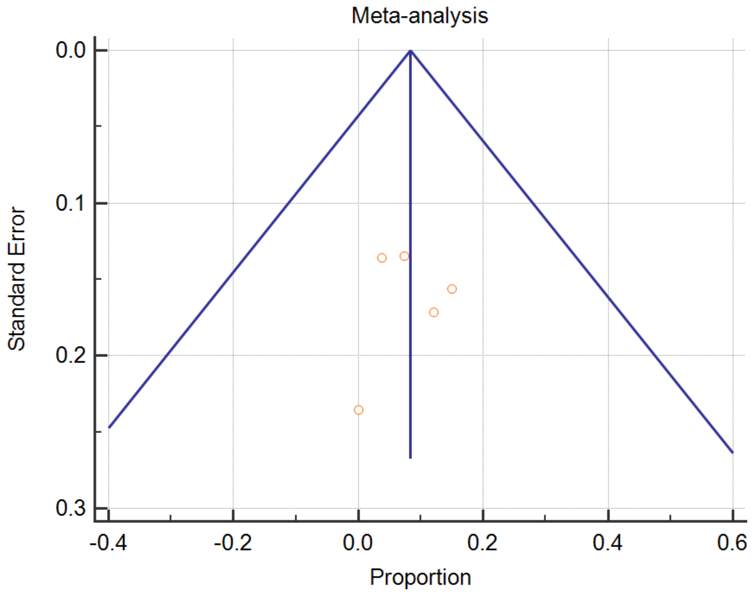

Figure 11: Funnel plot for TLR at 1 year in EVT RS group. Symmetrical

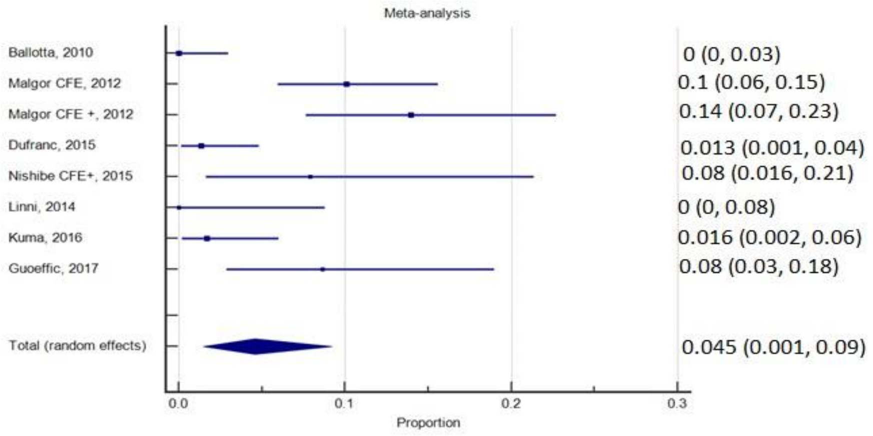

Figure 12: Forrest plot for TLR at 1 year in CFE group.

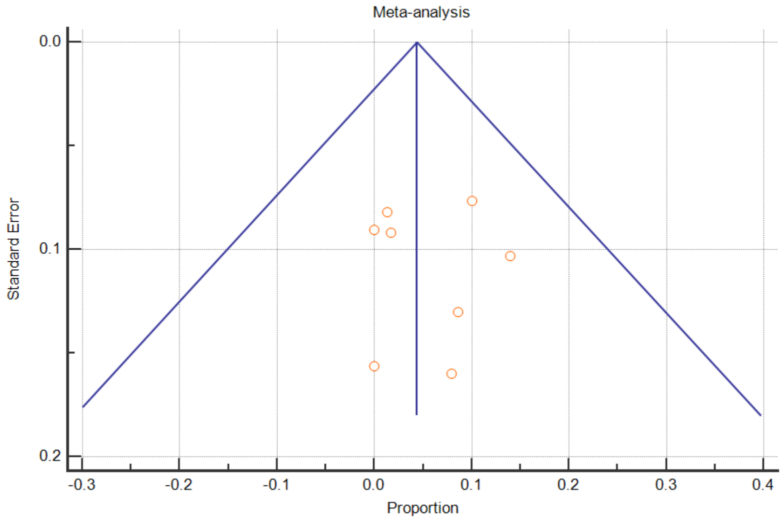

Figure 13: Funnel plot for TLR at 1 year in CFE group. Symmetrical

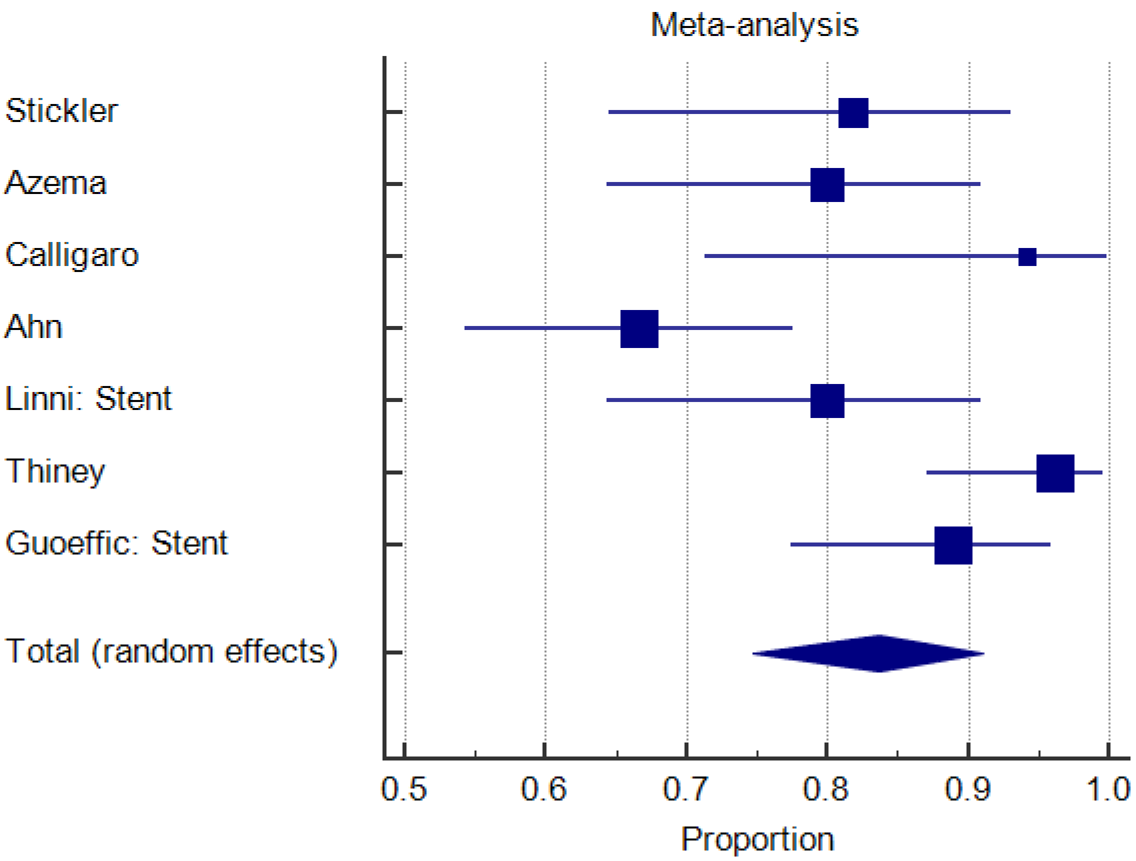

Figure 14: Forrest plot for PP at maximum follow up in EVT-RS. PP- 83.7% (95% CI 0.74-0.91).

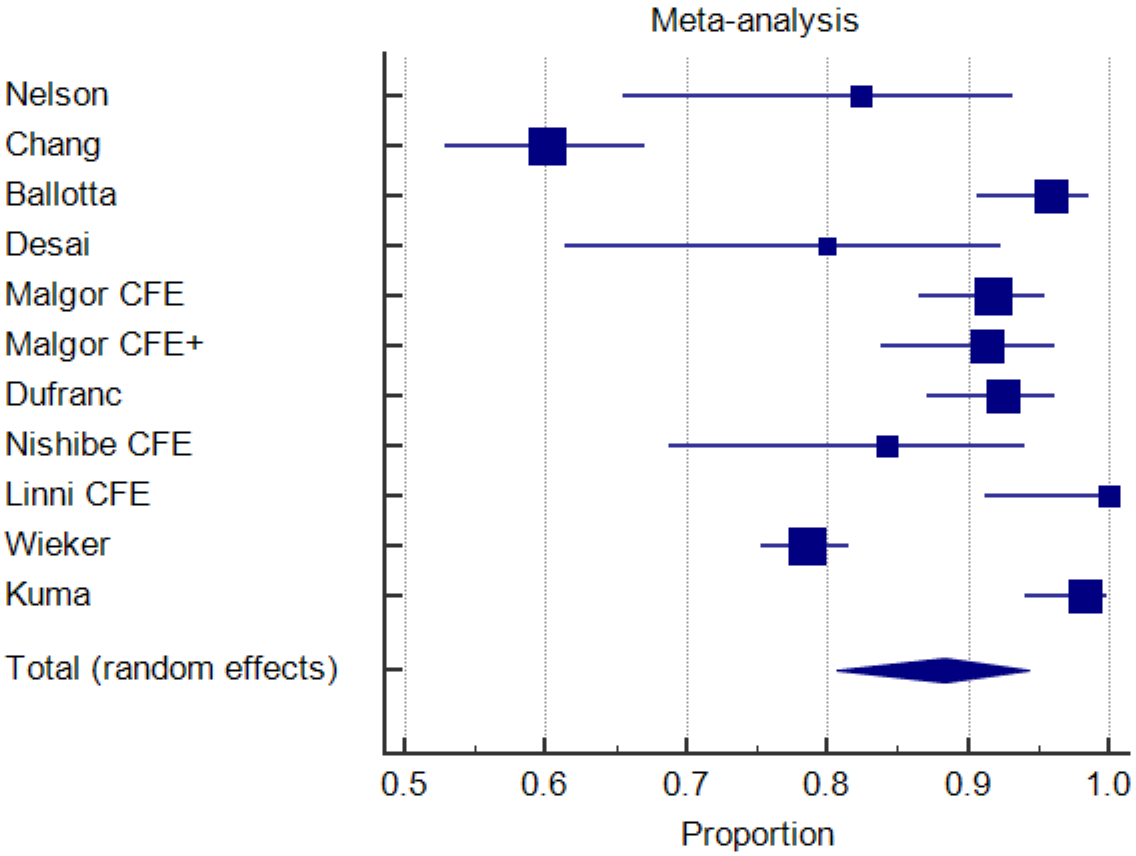

Figure 15: Forrest plot for PP in CFE at maximum follow up. PP- 88.34% (95% CI 0.81-0.94

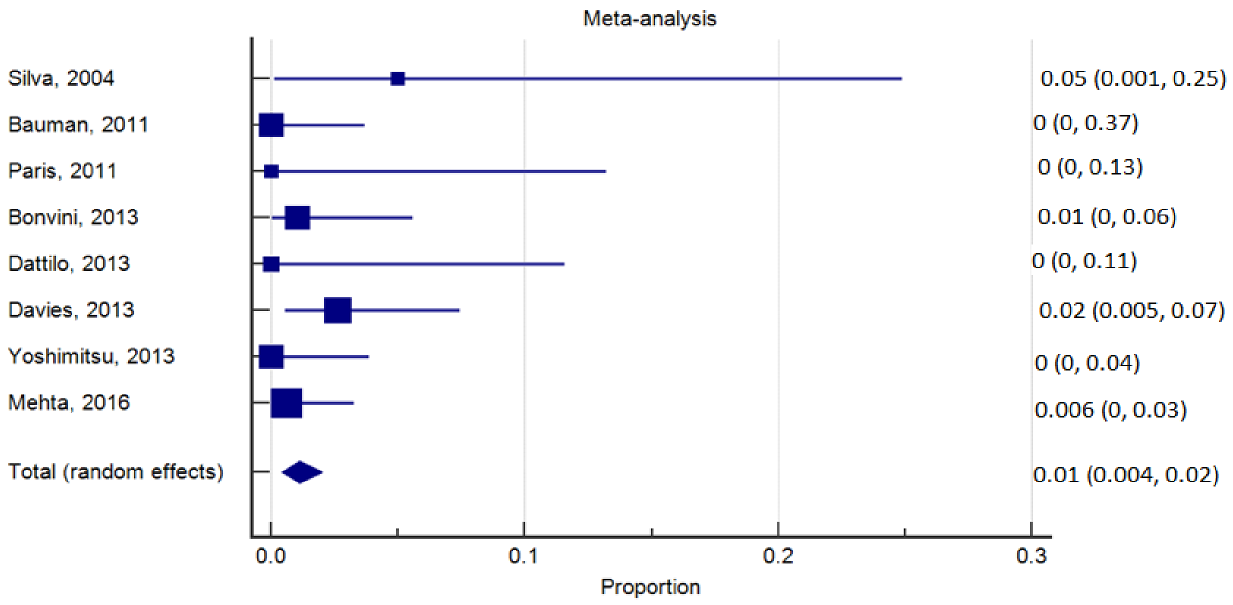

Figure 16: Forrest plot for mortality at 30 days in EVT SS group.

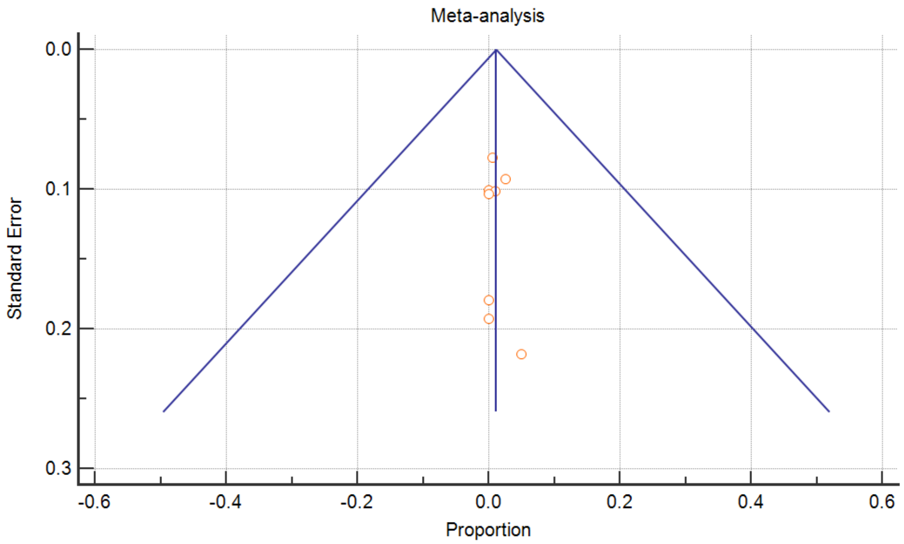

Figure 17: Funnel plot for mortality 30 days in EVT SS group . Symmetrical

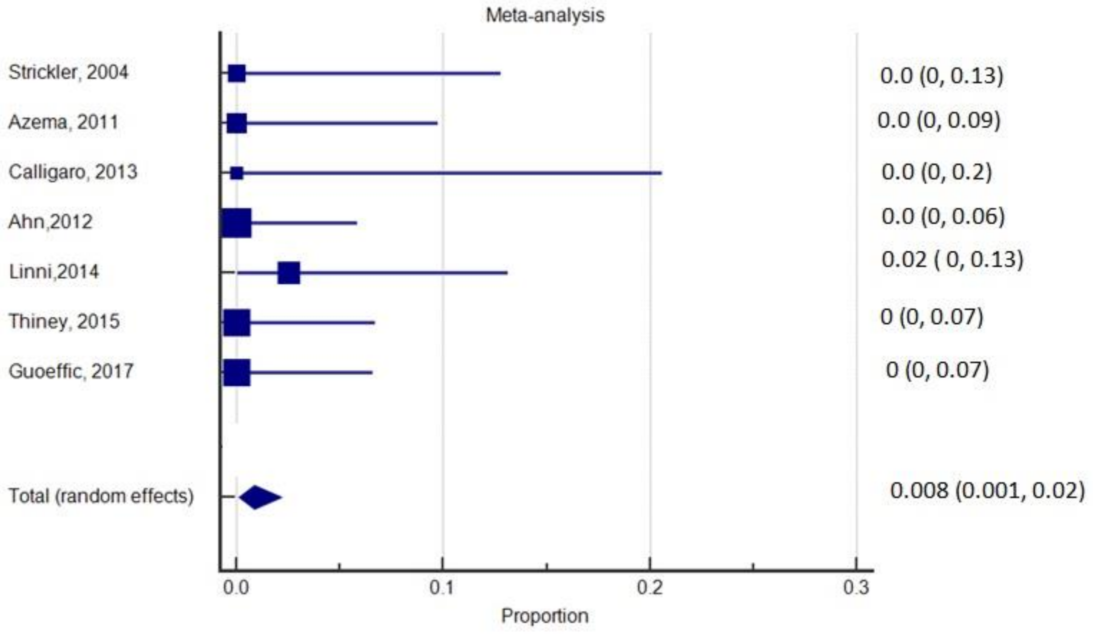

Figure 18: Forrest plot for Mortality 30 days in EVT RS group.

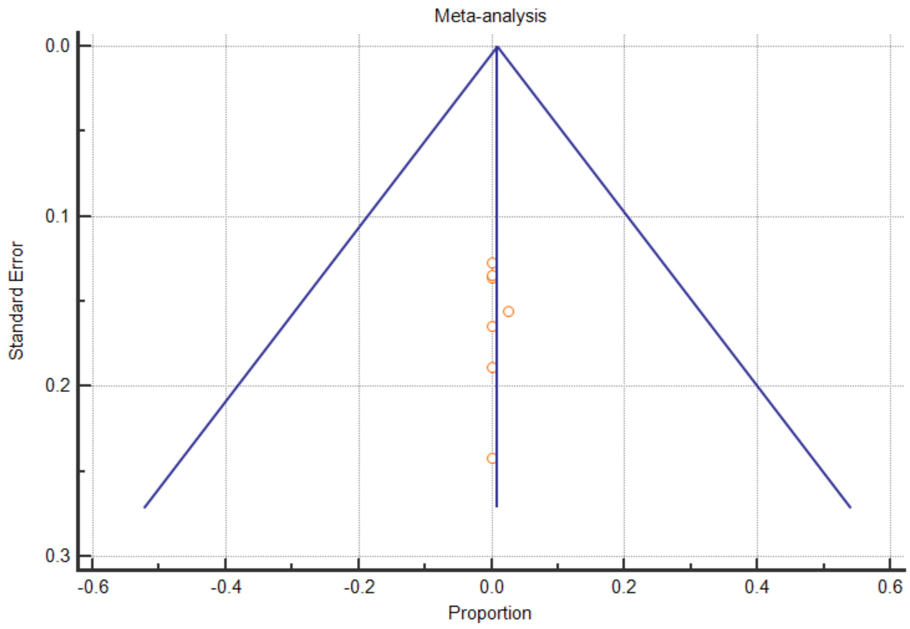

Figure 19: Funnel plot for Mortality at 30 days in EVT RS group. Symmetrical.

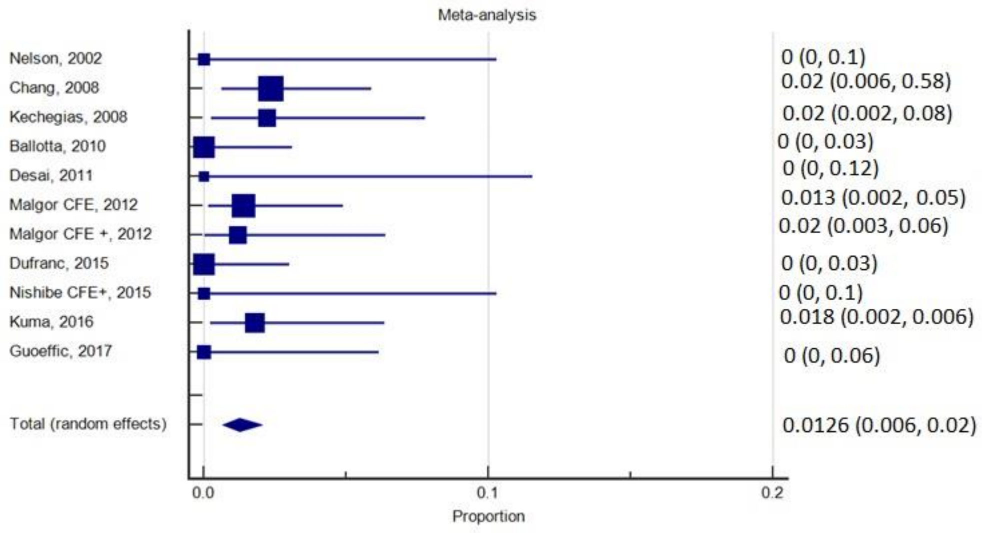

Figure 20: Forrest plot for mortality at 30 days in CFE group.

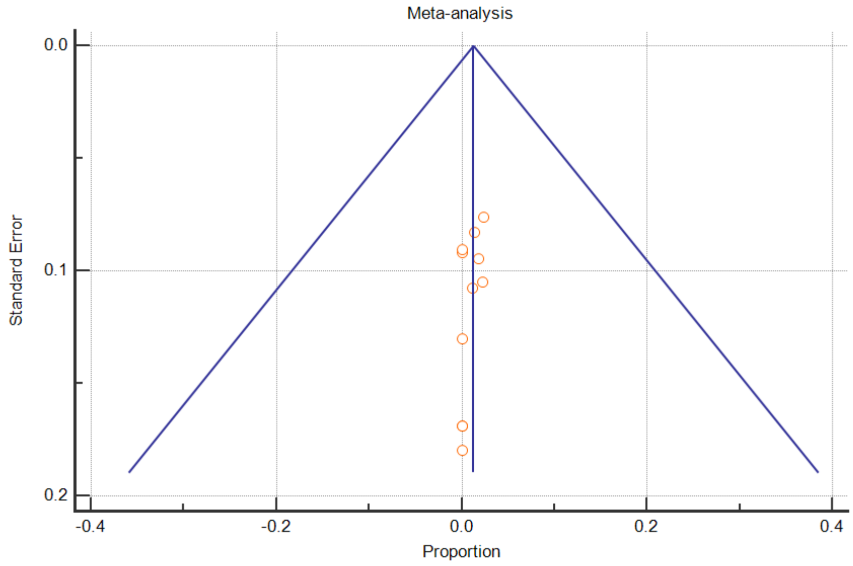

Figure 21: Funnel plot for mortality at 30 days in CFE group. Symmetrical.

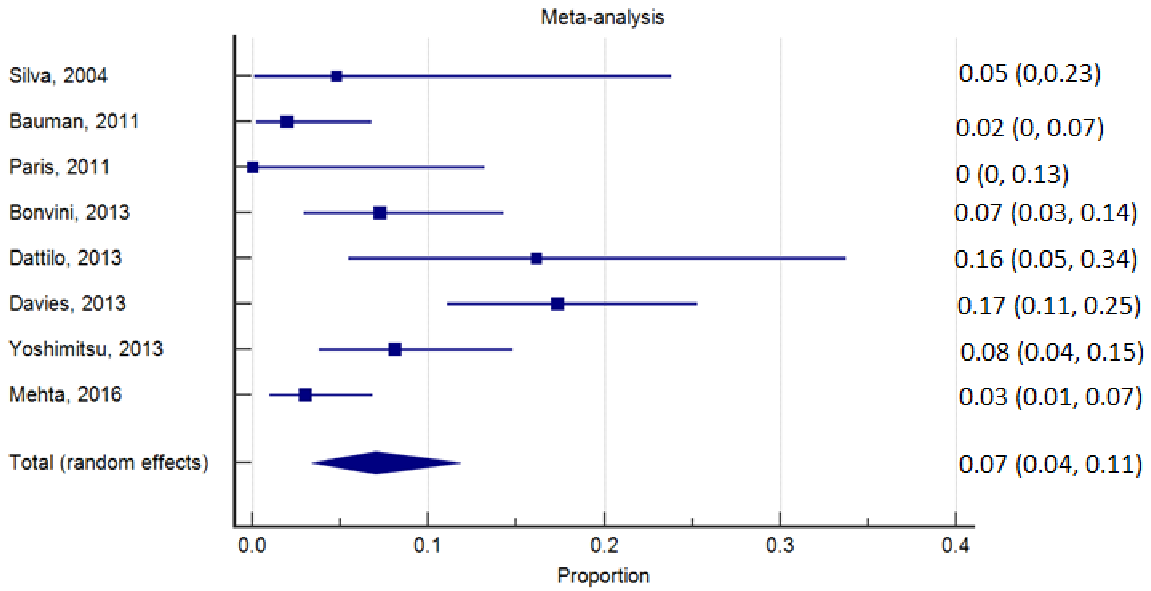

Figure 22: Forrest plot for local complications in EVTSS group.

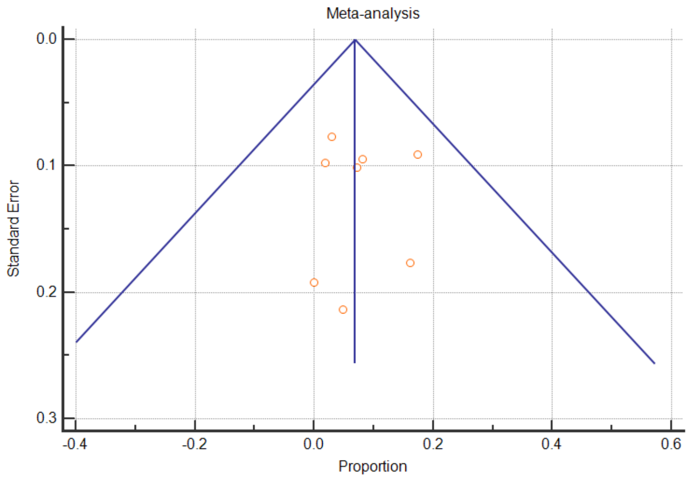

Figure 23: Funnel plot for local complications in EVT SS group. Symmetrical

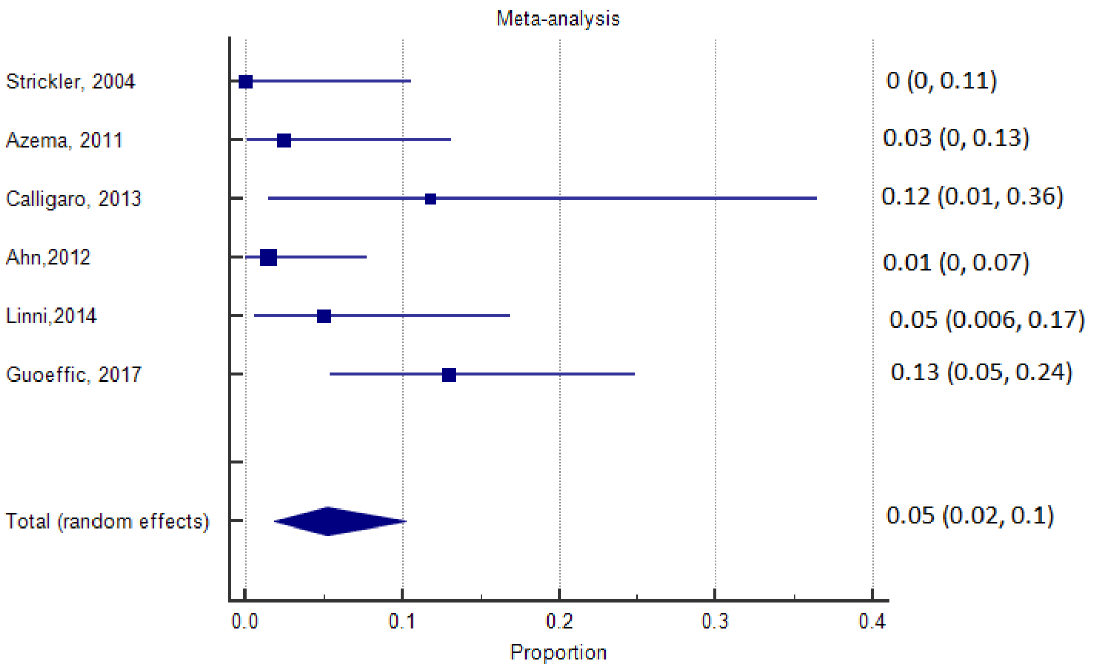

Figure 24: Forrest plot for local complications in EVT RS group.

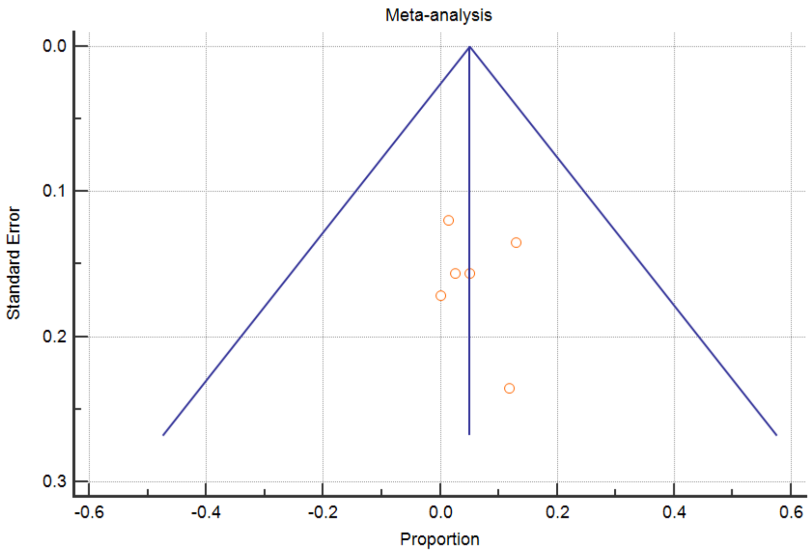

Figure 25: Funnel plot for local complications, EVTRS group. Symmetrical

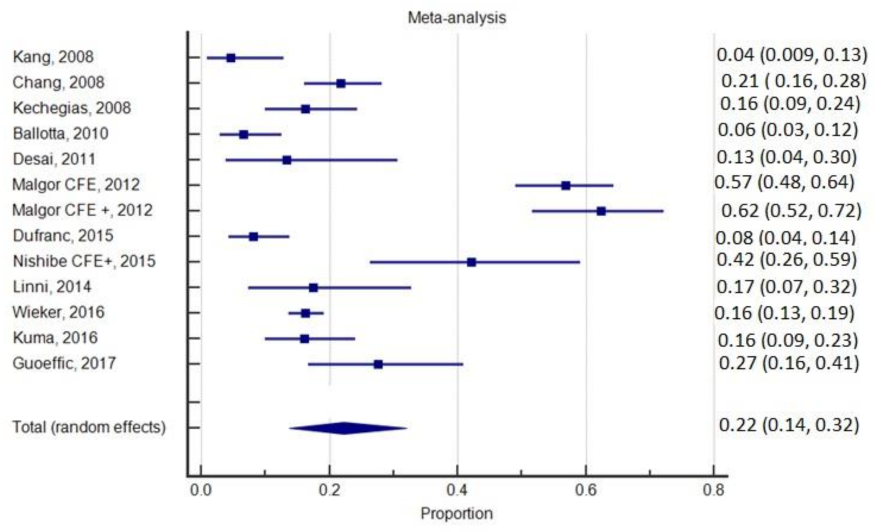

Figure 26: Forrest plot for local complications in CFE group.

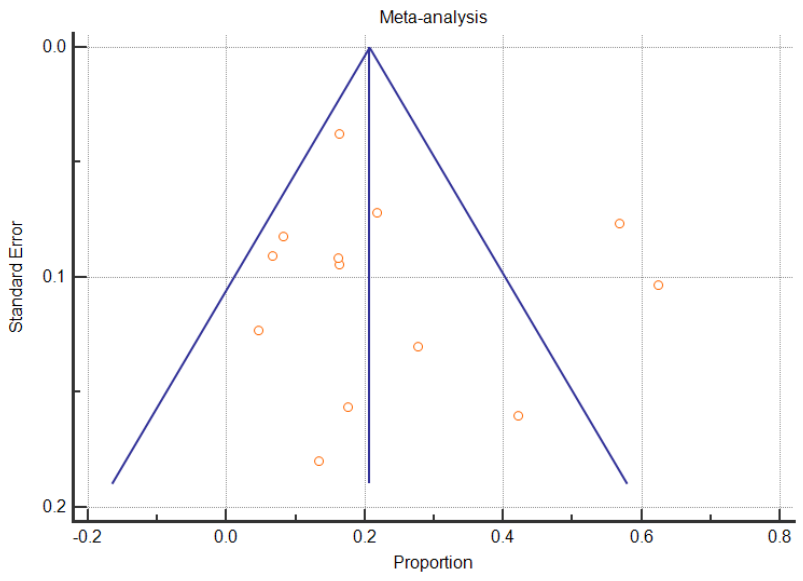

Figure 27: Funnel plot for Local complications in CFE group. Asymmetrical.

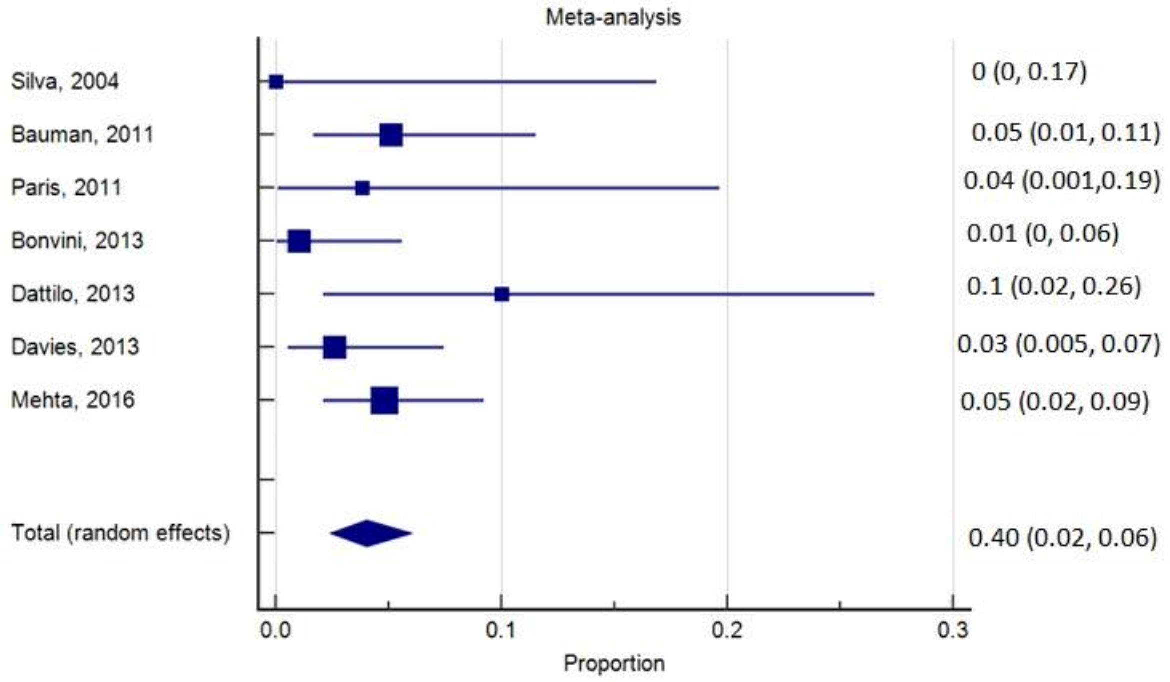

Figure 28: Forrest plot for Amputations in EVT SS group.

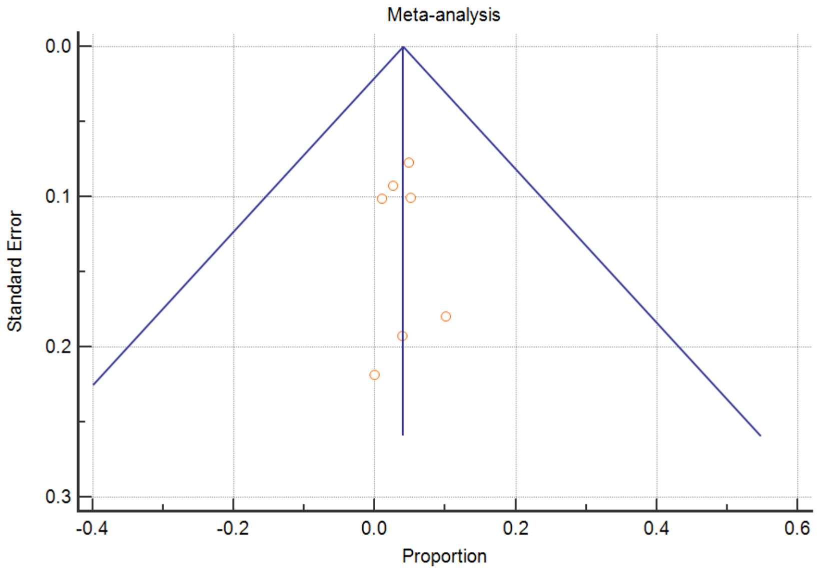

Figure 29: Funnel plot for Amputations in EVT SS group. Symmetrical

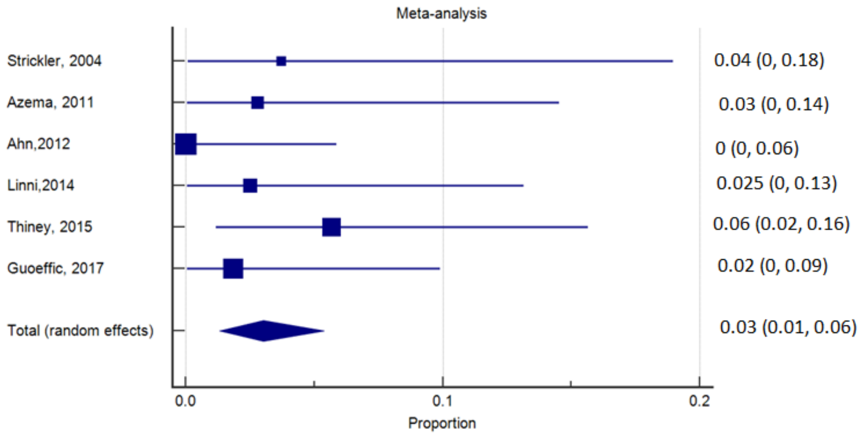

Figure 30: Forrest plot for amputations in EVT RS group.

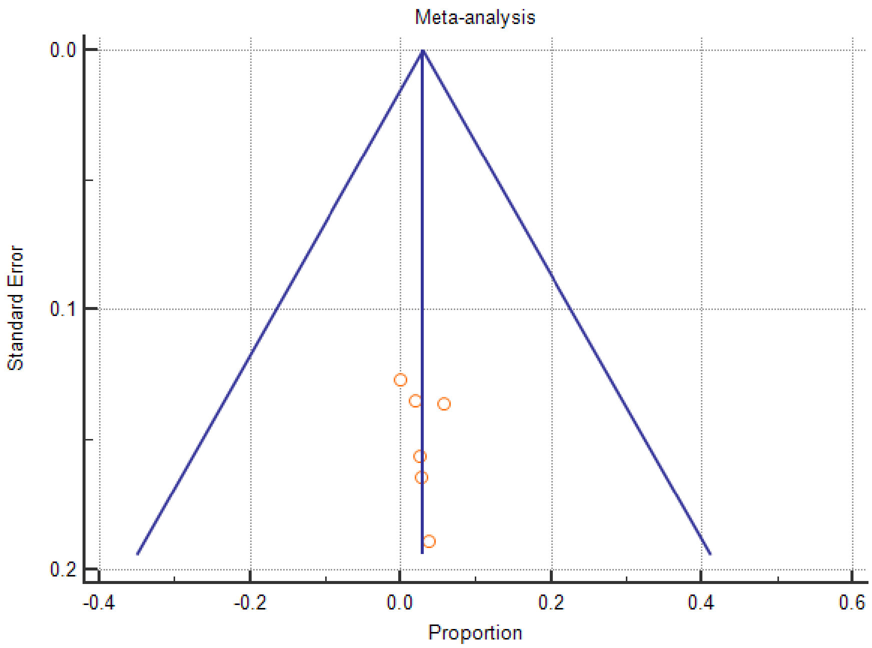

Figure 31: Funnel plot for amputations in EVT SS group. Symmetrical

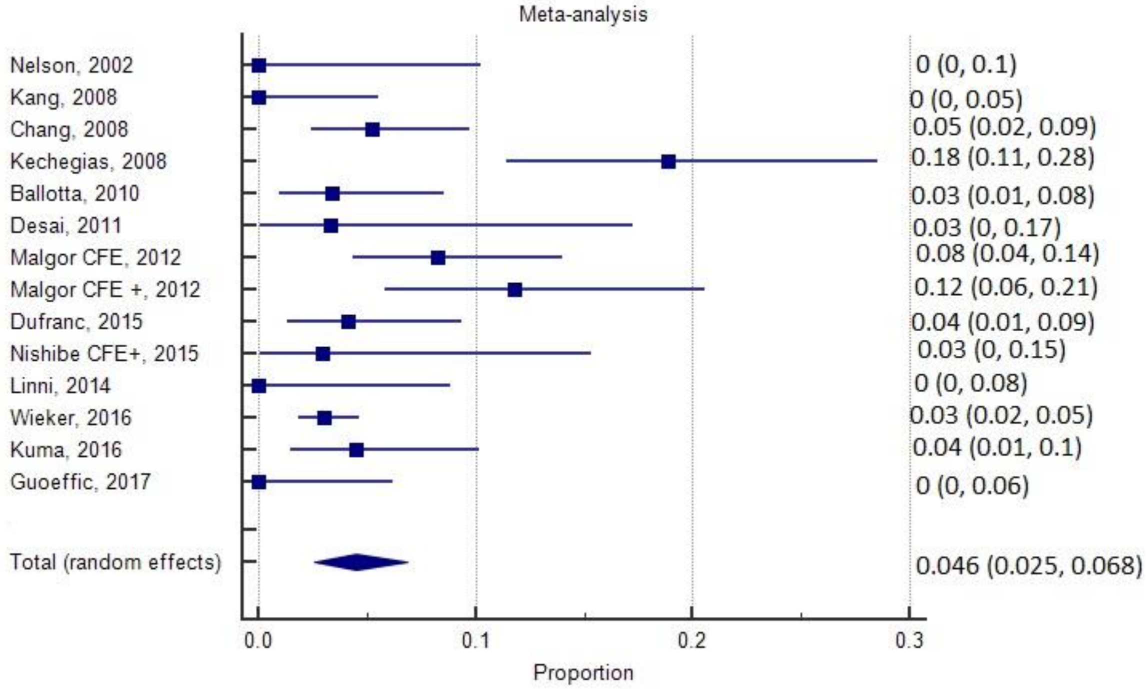

Figure 32: Forrest plot for amputations in CFE group.

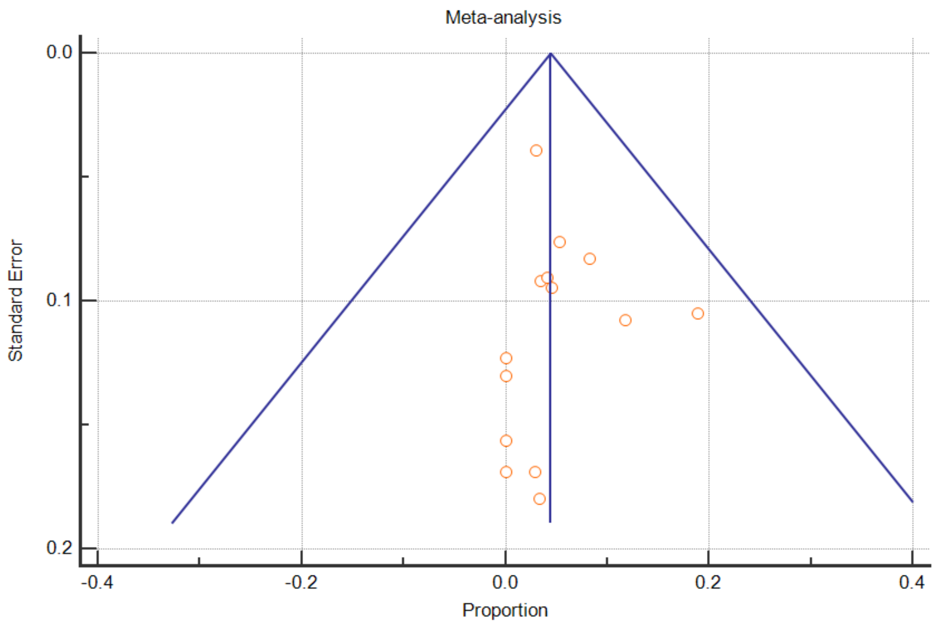

Figure 33: Funnel plot for amputations in CFE group. Asymmetrical.

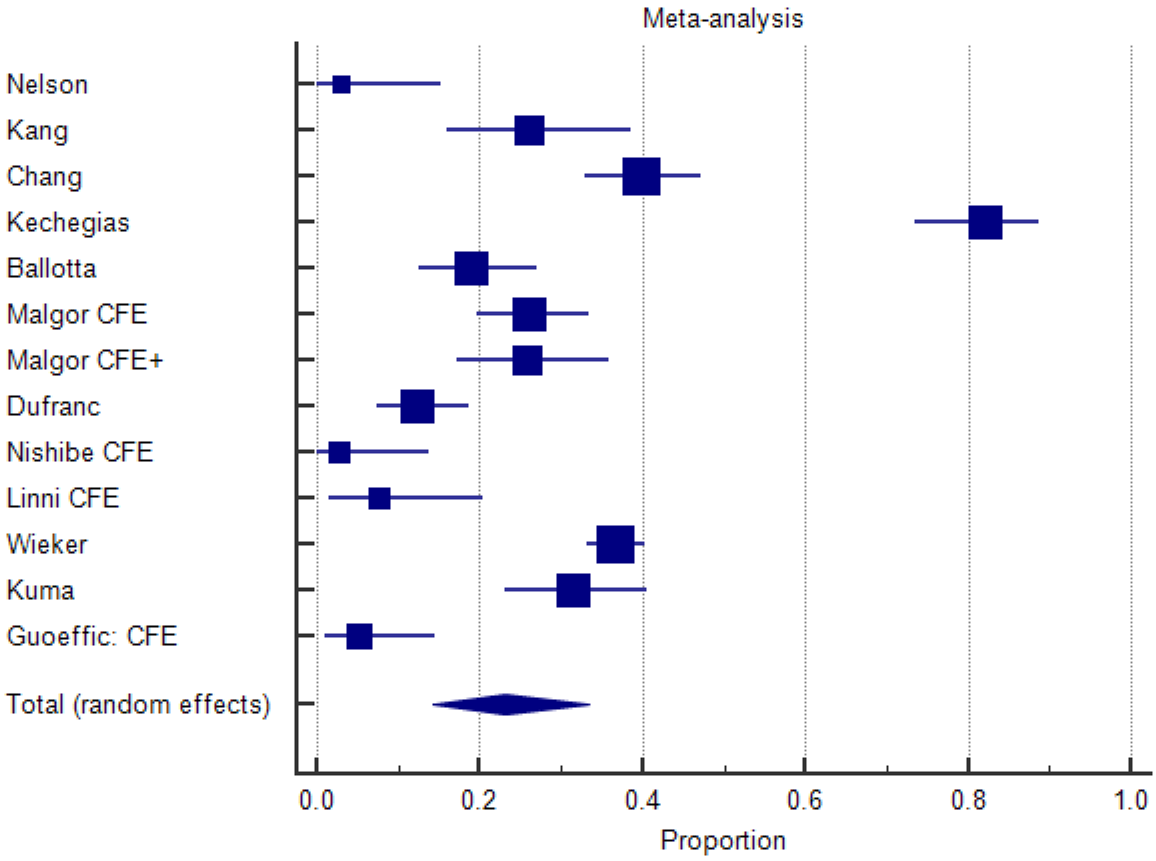

Figure 34: Forrest plot for mortality at max follow up in CFE group. Mortality was 23.1% (95% CI 0.14-0.33).

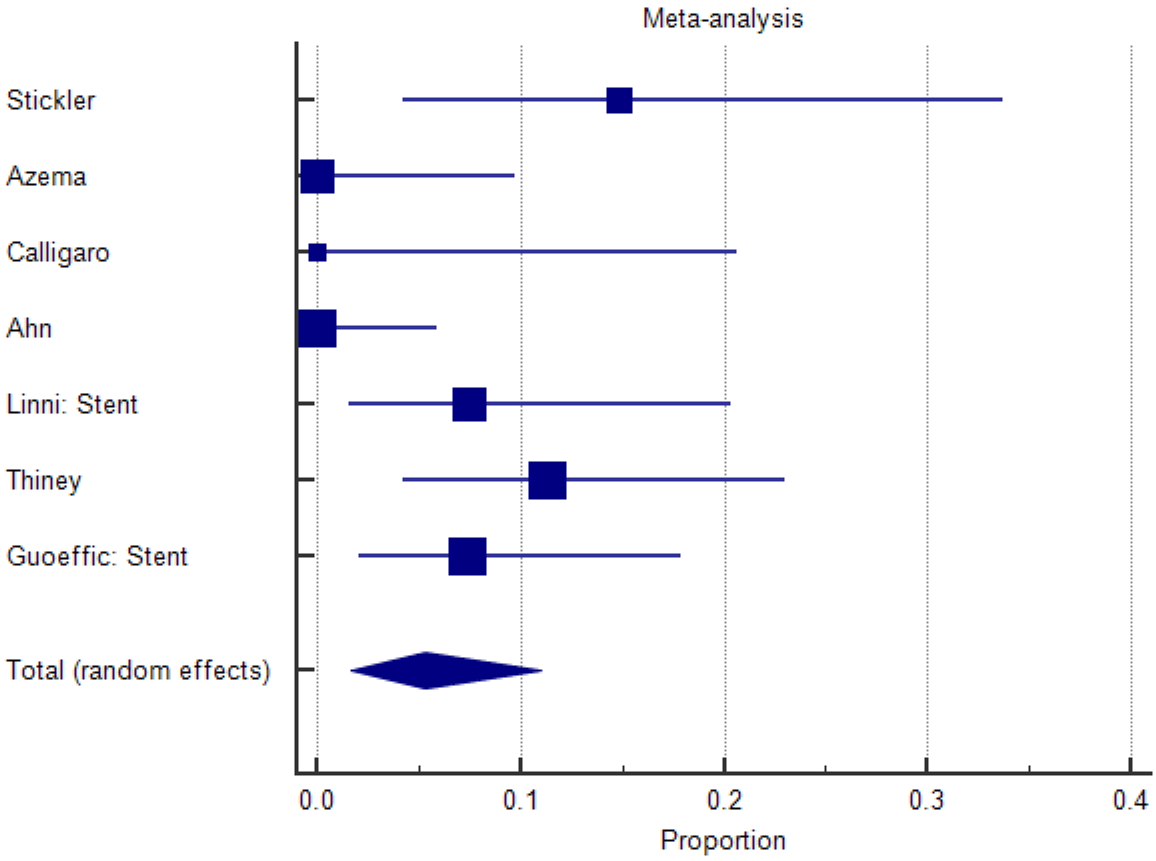

Figure 35: Forrest plot for mortality at max follow up in EVT-RS group. Mortality was 5.33% (95% CI 0.016-0.11)

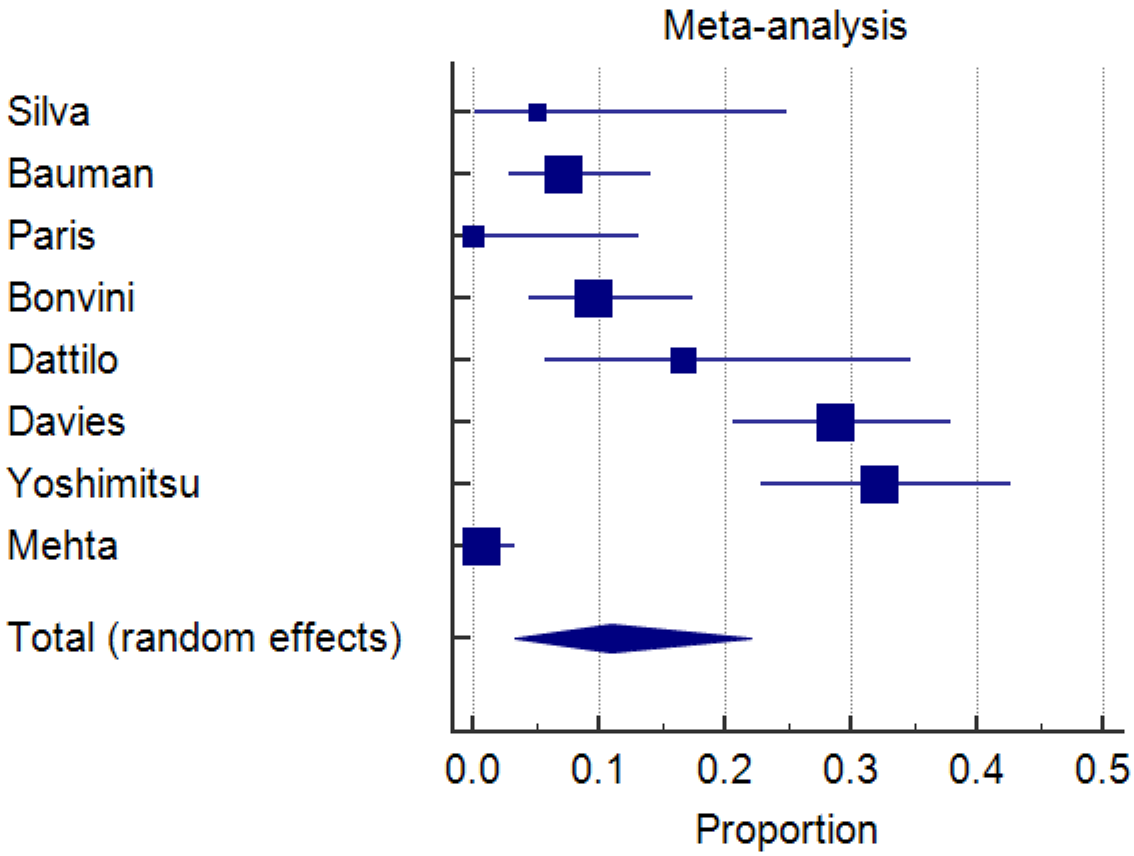

Figure 36: Forrest plot for mortality at max follow up in EVT-SS group. Mortality was 10.9% (95% CI 0.03-0.22).

Appendix 1:
Common femoral artery, Common femoral artery atherosclerotic disease, common femoral endarterectomy, common femoral artery stent, endovascular therapy common femoral artery, Peripheral arterial disease

Appendix 2

Definitions
'primary patency', defined as exempt from restenosis of the target lesion during follow-up. 'Primary assisted patency' is defined as patency of the target lesion following endovascular reintervention at the target vessel site in case of symptomatic restenosis. A reintervention at the index lesion site is defined as target lesion revascularization (TLR). If another lesion at the target vessel site but not the index lesion itself needs to be treated during follow-up this intervention is defined as target vessel revascularization (TVR).
